# Transfer Free Energies of Test Proteins Into Crowded Protein Solutions Have Simple Dependence on Crowder Concentration

**DOI:** 10.3389/fmolb.2019.00039

**Published:** 2019-05-29

**Authors:** Valery Nguemaha, Sanbo Qin, Huan-Xiang Zhou

**Affiliations:** ^1^Department of Physics and Institute of Molecular Biophysics, Florida State University, Tallahassee, FL, United States; ^2^Department of Chemistry and Department of Physics, University of Illinois at Chicago, Chicago, IL, United States

**Keywords:** macromolecular crowding, transfer free energy, excluded-volume, soft attraction, crowder concentration

## Abstract

The effects of macromolecular crowding on the thermodynamic properties of test proteins are determined by the latter's transfer free energies from a dilute solution to a crowded solution. The transfer free energies in turn are determined by effective protein-crowder interactions. When these interactions are modeled at the all-atom level, the transfer free energies may defy simple predictions. Here we investigated the dependence of the transfer free energy (Δμ) on crowder concentration. We represented both the test protein and the crowder proteins atomistically, and used a general interaction potential consisting of hard-core repulsion, non-polar attraction, and solvent-screened electrostatic terms. The chemical potential was rigorously calculated by FMAP (Qin and Zhou, 2014), which entails expressing the protein-crowder interaction terms as correlation functions and evaluating them via fast Fourier transform (FFT). To high accuracy, the transfer free energy can be decomposed into an excluded-volume component (Δμ_e−v_), arising from the hard-core repulsion, and a soft-attraction component (Δμ_s−a_), arising from non-polar and electrostatic interactions. The decomposition provides physical insight into crowding effects, in particular why such effects are very modest on protein folding stability. Further decomposition of Δμ_s−a_ into non-polar and electrostatic components does not work, because these two types of interactions are highly correlated in contributing to Δμ_s−a_. We found that Δμ_e−v_ fits well to the generalized fundamental measure theory (Qin and Zhou, 2010), which accounts for atomic details of the test protein but approximates the crowder proteins as spherical particles. Most interestingly, Δμ_s−a_ has a nearly linear dependence on crowder concentration. The latter result can be understood within a perturbed virial expansion of Δμ (in powers of crowder concentration), with Δμ_e−v_ as reference. Whereas the second virial coefficient deviates strongly from that of the reference system, higher virial coefficients are close to their reference counterparts, thus leaving the linear term to make the dominant contribution to Δμ_s−a_.

## Introduction

It is now well-recognized that “bystander” macromolecules in cellular milieus may significantly influence the biophysical properties of proteins (Zhou et al., 2008; Zhou, 2013; Gnutt and Ebbinghaus, 2016). Such influences can be detected by many experimental observables, including equilibrium sedimentation gradient (Rivas et al., 1999), protein folding and binding stability (Batra et al., 2009a,b; Miklos et al., 2011, 2013; Phillip et al., 2012; Wang et al., 2012; Sarkar et al., 2013), light scattering intensity (Wu and Minton, 2013), small-angle neutron scattering profile (Goldenberg and Argyle, 2014; Banks et al., 2018), and fluorescence resonance energy transfer (FRET) efficiency (Soranno et al., 2014). Theoretically these influences are determined by the transfer free energies of test proteins from a dilute solution to a solution of the macromolecular crowders (Minton, 1983; Zhou et al., 2008; Qin and Zhou, 2009; McGuffee and Elcock, 2010). Since the transfer free energies in turn are determined by the effective protein-crowder interactions, in principle the experimental data contain information about these intermolecular interactions. However, apart from the work of McGuffee and Elcock at “very significant computational expense” (McGuffee and Elcock, 2010), until recently it was only possible to use relatively crude models of protein-crowder interactions for calculating transfer free energies (Minton, 1981; Qin and Zhou, 2010; Kim and Mittal, 2013) or quantitatively modeling crowding effects (Cheung et al., 2005; Minh et al., 2006), thereby limiting our ability to interpret and fully utilize the experimental data. To mitigate this problem, an FFT-based method for Modeling Atomistic Protein-crowder interactions, or FMAP, has been developed (Qin and Zhou, 2013, 2014). Most recently FMAP was used to quantitatively interpret FRET efficiency data for disordered proteins in the presence of polyethylene glycol (Soranno et al., 2014), implicating mild attraction between the test proteins and the polymer crowder (Nguemaha et al., 2018). Here we used FMAP to calculate the transfer free energy (Δμ) of folded and unfolded test proteins from dilute to crowded protein solutions, paying particular attention to the dependence of Δμ on crowder concentration. Even with an all-atom representation for both the test and the crowder proteins, the dependence of Δμ on crowder concentration was found to follow simple relations. We explore the physical reasons for this simple behavior.

As in our previous study (Qin and Zhou, 2014), we assumed a general, implicit-solvent energy function for protein-crowder interactions, consisting of hard-core steric repulsion, non-polar attraction, and solvent-screened electrostatic terms:

(1) $$\begin{array}{l} U_{\text{int}}=U_{\text{st}}+U_{\text{n}-\text{a}}+U_{\text{elec}} \end{array}$$

Whenever *r*_*ij*_, the distance of any pair of protein-crowder atoms, is less than the sum of their hard-core radii, (σ_*ii*_ + σ_*jj*_)/2, the steric term *U*_st_ goes to ∞. When the test protein is free of such steric clashes with crowder atoms, *U*_st_ vanishes and the two soft interaction terms come into play. Specifically, the non-polar attraction has the form of a Lennard-Jones potential:

(2) $$\begin{array}{l} U_{\text{n}-\text{a}}=\underset{ij}{\sum }4\varepsilon_{ij}\left[{\left(\sigma_{ij}/r_{ij}\right)}^{12}-{\left(\sigma_{ij}/r_{ij}\right)}^{6}\right]=\underset{ij}{\sum }\left(A_{ij}/r_{ij}^{12}-B_{ij}/r_{ij}^{6}\right) \end{array}$$

where ε_*ij*_ is the magnitude of the non-polar attraction between the *i*-*j* pair of atoms. The solvent-screened electrostatic term has the form of a Debye-Hückel potential:

(3) $$\begin{array}{l} U_{\text{elec}}=\underset{ij}{\sum }q_{i}q_{j}\exp\left(-r_{ij}/\lambda \right)/\kappa r_{ij} \end{array}$$

where *q*_*i*_ are atomic charges, and λ and κ are the Debye screening length and the dielectric constant, respectively, of the crowder solution.

FMAP finds the transfer free energy from an average of the Boltzmann factor of the protein-crowder interaction energy (Qin and Zhou, 2013, 2014)

(4) $$\begin{array}{l} \exp\left(-\Delta \mu /k_{\text{B}}T\right)=<\exp\left[-U_{\text{int}}\left(\text{R}\right)/k_{\text{B}}T\right]{>}_{\text{R},\Omega ,\text{c}} \end{array}$$

More specifically, the test protein is fictitiously placed into the crowder solution, and the average is taken over the position (“**R**”) and orientation (“**Ω**”) of the test protein and configuration (“**c**”) of the crowders. The average over **R** is taken care of by FFT, and is the core component of FMAP. The average over **Ω** and **c** is realized by repeating MFAP calculations over different orientations of the test protein and different configurations of the crowder solution.

Here we obtained the transfer energies of eight test proteins over a wide range of concentrations of two crowder proteins. There are two main findings. First, the transfer free energy can be accurately decomposed into an excluded-volume component (Δμ_e−v_), arising from the hard-core repulsion, and a soft-attraction component (Δμ_s−a_), arising from non-polar and electrostatic interactions. Second, whereas the excluded-volume component has a complex dependence on crowder concentration, the soft-attraction component has a nearly linear dependence on crowder concentration. We explain this interesting result by a perturbed virial expansion of Δμ.

## Computational Methods

**Figure 1:**
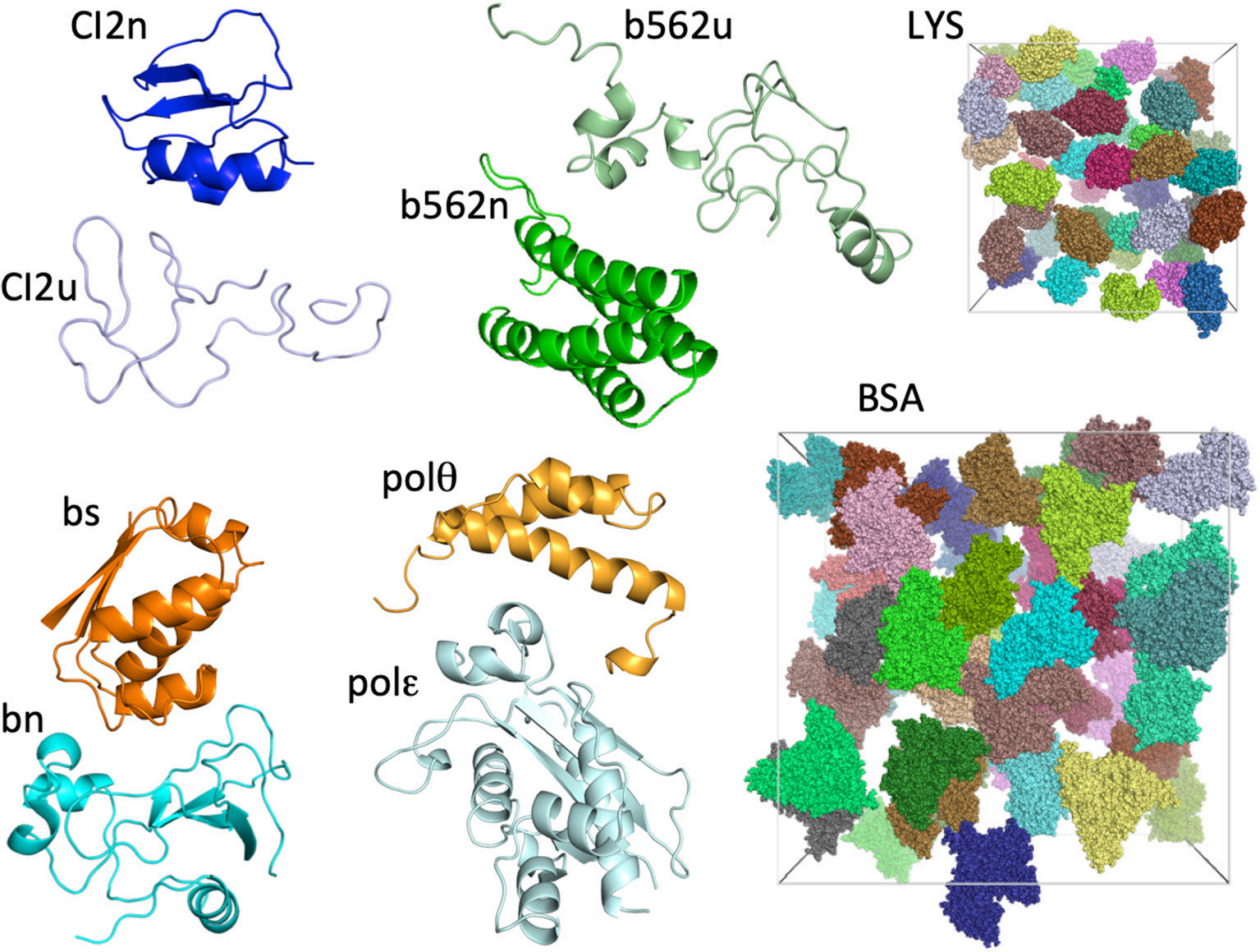
The eight test proteins and two crowder proteins in the present study. For the two crowder proteins, 42 copies are present in cubic boxes with side lengths of 174 and 300 Å, resulting in concentrations of 217 mg/mL for LYS and 196 mg/mL for BSA.

The eight test proteins studied are: native and unfolded chymotrypsin inhibitor 2 (CI2n and CI2u, respectively), native and unfolded cytochrome b562 (b562n and b562u, respectively), barnase (bn), barstar (bs), and the DNA polymerase III θ and ε subunits (polθ and polε, respectively). As in our previous study (Qin and Zhou, 2014), we represented each protein by a single conformation (Figure 1). The two crowder proteins are lysozyme (LYS) and bovine serum albumin (BSA) (Figure 1).

To obtain the crowder protein configurations, first hard-sphere simulations were carried out and then the hard spheres in the final snapshots were replaced by randomly orientated protein molecules. The simulations were run using a C++ code (https://cims.nyu.edu/~donev/Packing/C++/), written by Skoge et al. (Skoge et al., 2006). In short, *N* spheres in a cubic box were grown from points at a steady rate and underwent ballistic collisions. The box had a side length of 1 and periodic boundary conditions were imposed. The simulations were terminated when the hard spheres grew to a desired radius. Specifically, for the simulations intended for LYS, the final radius was 0.1485, such that the hard-sphere volume fraction at *N* = 48 reached 0.658; for BSA, the final radius was 0.14 and the volume fraction at *N* = 48 was 0.552. Ten replicate simulations were run at each *N* for replacement into each of the two crowder proteins.

For replacing the hard spheres by protein molecules, the radii of the spheres were scaled to appropriate lengths to allow for the spheres to enclose the proteins. For the simulations intended for LYS, the unit length of the simulation box was scaled to 174 Å, and so the spheres were mapped to a radius of 25.84 Å. For BSA, the corresponding simulation box was scaled to a 300 Å side length, leading to a hard sphere radius of 42.0 Å. These spheres were sufficiently large to enclose the vast majority of the atoms in each crowder protein. The spheres were replaced by protein molecules one at a time. The protein molecules were assigned random orientations, by choosing a random direction for a unit vector attached to the protein and rotating the protein around the unit vector by a random angle between 0 and 360° (Qin et al., 2011). When placing a new protein molecule, random orientations were repeatedly chosen until it did not clash with any of the protein molecules already placed (including their periodic images). The threshold for clash was 4.0 Å for any interatomic distance between two protein molecules. This process was repeated until all the hard spheres in the simulation box were successful replaced by protein molecules. The number, *N*, of crowder molecules in the simulation boxes ranged from 6 to 48, in increments of 6. At the highest number, the crowder concentrations were 217 mg/mL for LYS and 196 mg/mL for BSA.

FMAP entails fictitiously placing a test protein into the crowder box and calculating the interaction energy between the test protein and the crowder proteins. The interaction energy function is given by equations (1)–(3), and the parameters are those described in our previous study. Specifically, the Lennard-Jones parameters were taken from Autodock (Morris et al., 2009) and the partial charges were taken from Amber (Cornell et al., 1995). To achieve a better balance between *U*_n - a_ and *U*_elec_ (as judged by, e.g., salt and temperature dependences of second virial coefficients of proteins in unpublished work), we scaled down the former by a factor of 0.2 (for comparison, Autodock applied a scaling factor around 0.16), and scaled up the latter by a factor of 2.0. The temperature was 298 K (where the solvent dielectric constant was 78.4) and the ionic strength was 0.15 M.

At each crowder concentration, 10 independent configurations of crowders were generated; for each crowder configuration, 500 random orientations for each test protein were chosen. So altogether 5,000 FMAP calculations were carried out for each test protein at each crowder concentration, and the results were averaged to yield the transfer free energy. To test the additivity between the excluded-volume component and the soft-attraction component, we also carried out corresponding averaging to obtain these components (see below).

Two methods were used to do error analysis. The first was bootstrap. Here the 5,000 individual values of the transfer free energy (or a component thereof) were pooled to create the original sample. Bootstrap samples (with the same size, 5,000, as in the original sample) were then generated by randomly drawing from the original sample, and the standard deviation of the data in each bootstrap sample was calculated. The error of the FMAP calculation was finally estimated as the mean of the standard deviations of 10,000 bootstrap samples. The second was the block decorrelation technique of Flyvbjerg and Petersen (1989) (code downloaded from https://github.com/manoharan-lab/flyvbjerg-std-err/). Here we treated the 5,000 data points as a time series. These data points were “blocked” in successive generations. Specifically, the data points in the first generation were the original ones; in the second generation, the first two data points, the next two data points, and so on were each “blocked,” i.e., merged and replaced by their averages. This blocking process continued until the total number of blocked data points went below a cutoff of 15. At each generation, the variance of the blocked data points was calculated. The variance reached a plateau before the cutoff, and the square root of the plateau value was taken as the error estimate.

## Results

### Additivity Between Excluded-Volume and Soft-Attraction Components

As shown by equation (4), the transfer free energy Δμ is given by the average of the Boltzmann factor of the protein-crowder interaction energy *U*_int_; the average needs to be taken over the position **R** of a fictitious placement of the test protein into the crowder box, the orientation **Ω** of the test protein, and the configuration **c** of the crowders. For a given **Ω** and a given **c**, FMAP calculates the average over **R** from values of *U*_int_ at grid points within the crowder box. The grid points can be separated into ones with protein-crowder clash and ones that are clash-free. Note that exp(−*U*_st_/*k*_B_*T*) has value 0 at the clashed grid points and value 1 at the clash-free ones; the two soft interactions only operate at the clash-free grid points. Based on these considerations, we can write the average of exp(−*U*_int_/*k*_B_ *T*) over **R** as

(5) $$\begin{array}{l} <\exp\left(-U_{\text{int}}/k_{\text{B}}T\right){>}_{\text{R}}=<\exp\left(-U_{\text{st}}/k_{\text{B}}T\right){>}_{\text{R}}\\ \times <\exp\left[-\left(U_{\text{n}-\text{a}}+U_{\text{elec}}\right)/k_{\text{B}}T\right]{>}_{1} \end{array}$$

where < ⋯>_**R**_ and < ⋯>_1_ signify averaging over all the grid points and clash-free ones, respectively. Note that < exp(−*U*_st_/*k*_B_*T*) >_**R**_ is simply the clash-free fraction of grid points. Corresponding to the factorization in equation (5), we can write the transfer free energy, calculated without averaging over **Ω** and **c**, as the sum of an excluded-volume component and a soft-attraction component:

(6) $$\begin{array}{l} \Delta \mu =\Delta \mu_{\text{e}-\text{v}}+\Delta \mu_{\text{s}-\text{a}},\text{for a single }\Omega \text{and a single}c \end{array}$$

The excluded-volume component is given by the clash-free fraction,

(7) $$\begin{array}{l} \exp\left(-\Delta \mu_{\text{e}-\text{v}}/k_{\text{B}}T\right)=<\exp\left(-U_{\text{st}}/k_{\text{B}}T\right){>}_{\text{R}} \end{array}$$

whereas the soft-attraction component is given by the combined soft interactions at the clash-free grid points:

(8) $$\begin{array}{l} \exp\left(-\Delta \mu_{\text{s}-\text{a}}/k_{\text{B}}T\right)=<\exp\left[-\left(U_{\text{n}-\text{a}}+U_{\text{elec}}\right)/k_{\text{B}}T\right]\;{>}_{1} \end{array}$$

**Figure 2:**
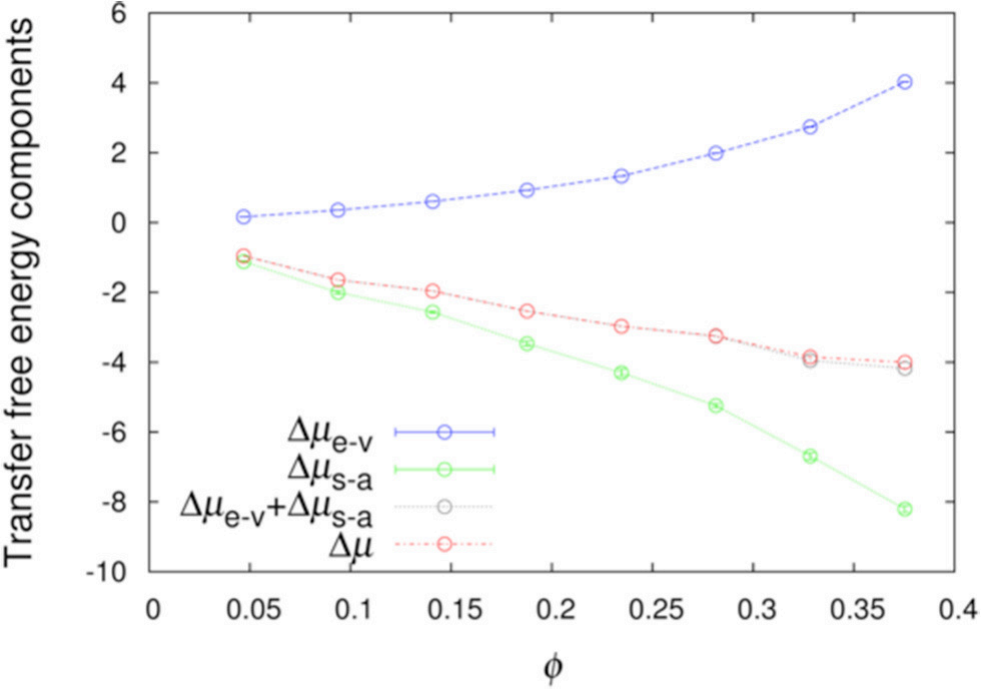
Additivity of the excluded-volume and soft-attraction components of the transfer free energy.

We further averaged < exp(−*U*_int_/*k*_B_*T*) >_**R**_ over combinations of 500 test protein orientations and 10 crowder configurations to obtain the transfer free energy Δμ. Specifically, the algebraic average of 5,000 individual values of < exp(−*U*_int_/*k*_B_*T*) >_**R**_ was calculated and then converted to Δμ. Similarly, we averaged < exp(−*U*_st_/*k*_B_*T*) >_**R**_ and < exp[−(*U*_n−a_+*U*_elec_)/*k*_B_*T*] >_1_ over the 5,000 **Ω/c** combinations to obtain Δμ_e−v_ and Δμ_s−a_, respectively. The sum of Δμ_e−v_ and Δμ_s−a_ provides a very accurate estimate of Δμ (Figure 2), demonstrating the additivity of these two components. The errors reported by two methods are very similar (Figure S1), and hence in Figure 2 and hereafter we only show errors determined by the bootstrap method.

If we have an infinitely large crowder box, then its different regions give a good representation for the configurations of a finite crowder box. Likewise, when the test protein with a single orientation is fictitiously placed into different regions of the infinite crowder box, it is as if many different orientations of the test protein are probed by a finite crowder box. Hence, for an infinitely large crowder box, a separate average over crowder configurations and test protein orientations is unnecessary; then the separation of Δμ into Δμ_e−v_ and Δμ_s−a_ is exact. That our results accurately conform to additivity provides an indication that our crowder box is sufficiently large. In particular, the clash-free fraction, < exp(−*U*_st_/*k*_B_*T*) >_**R**_, is highly constant among the 5,000 **Ω/c** combinations, as indicated by very small Δμ_e−v_ errors (< 0.01 kcal/mol, except for polε in the most concentrated LYS solution, where the error is 0.02 kcal/mol; see also below) (Figure 2). The decomposition into Δμ_e−v_ and Δμ_s−a_ provides physical insight into the transfer free energy. Δμ_e−v_ is necessarily positive, whereas Δμ_s−a_ can be expected to be negative. As Figure 2 shows, these two quantities largely cancel each other, leading to a relatively modest magnitude for Δμ.

We also asked whether Δμ_s−a_ could be further decomposed into the separate contributions of the two types of soft interactions. To that end, we carried out the averages of exp(−*U*_n−a_/*k*_B_*T*) and exp(−*U*_elec_/*k*_B_*T*), i.e., by including only one of the two types of soft interactions. For convenience we refer to the corresponding chemical potentials as Δμ_n−a_ and Δμ_elec_, respectively. As shown in Figure S2, the magnitude of μ_s−a_ is much larger than the sum of Δμ_n−a_ and Δμ_elec_, indicating strong correlations between the two types of soft interactions. Indeed, one expects that the strongest electrostatic attractions occur when the test protein is apposed to crowder proteins with high charge and shape complementarity, but high shape complementarity also leads to strong non-polar attraction.

For the largest test protein, polε, in the most concentrated LYS solution (217 mg/mL), the clash-free fraction in 2,086 of the 5,000 **Ω/c** combinations was 0, i.e., not a single grid point was clash-free. In this concentrated crowder solution, the probability that voids large enough to accommodate polε is very small, which explains the high percentage of fully clashed **Ω/c** combinations as well as the relatively higher error of Δμ_e−v_ (calculated on the 2,914 **Ω/c** combinations with clash-free grid points). The Δμ_e−v_ value thus calculated was corrected by adding −*k*_B_*T*ln(2, 914/5, 000) to account for the fully clashed **Ω/c** combinations. The same correction also applies to ΔΔμ in this case. Values of Δμ, μ_e−v_, and Δμ_s−a_ for the eight test proteins are presented in Table S1 for LYS crowding and Table S2 for BSA crowding.

### Theoretical Modeling of Excluded-Volume Component

For calculating the excluded-volume transfer free energy, scaled-particle and other theories have been developed for test particles and crowder particles that have spherical and other simple shapes. Our generalized fundamental measure theory (GFMT) has enabled the test proteins to be represented at the all-atom level, though crowders still have to be modeled as spheres (Qin and Zhou, 2010). GFMT predicts the excluded-volume component as

(9) $$\begin{array}{l} \Delta \mu_{\text{e}-\text{v}}=\Pi_{\text{c}}v_{\text{p}}+\gamma_{\text{c}}s_{\text{p}}+\kappa_{\text{c}}l_{\text{p}}-k_{\text{B}}T\ln\left(1-\phi \right) \end{array}$$

where *v*_p_, *s*_p_, and *l*_p_ are the volume, surface area, and integrated mean curvature (with dimension of length) of the test protein; Π_c_ is the osmotic pressure of the crowder solution, and γ_c_ and κ_c_ are the corresponding quantities for surface tension and bending rigidity; and ϕ is the total volume fraction of the crowders. The latter is given by ϕ = *V*_*c*_ρ_*c*_, where *V*_c_ and ρ_c_ are the volume and number density of the crowders. Two other quantities, ρ_R_ = *R*_c_ρ_c_ and ρ_S_ = *S*_c_ρ_c_, with *R*_c_ and *S*_c_ denoting the radius and surface area of the crowders, are needed to define Π_c_, γ_c_, and κ_c._ The results are

(10) $$\begin{array}{l} \frac{\Pi_{\text{c}}}{k_{\text{B}}T}=\frac{\rho }{1-\phi }+\frac{\rho_{\text{R}}\rho_{\text{S}}}{{\left(1-\phi \right)}^{2}}+\frac{{\rho_{\text{S}}}^{3}}{{12\pi \left(1-\phi \right)}^{3}} \end{array}$$

(11) $$\begin{array}{l} \frac{\gamma_{\text{c}}}{T}=\frac{\rho_{\text{R}}}{1-\phi }+\frac{{\rho_{\text{S}}}^{2}}{{8\pi \left(1-\phi \right)}^{2}} \end{array}$$

(12) $$\begin{array}{l} \frac{\kappa_{\text{c}}}{k_{\text{B}}T}=\frac{\rho_{\text{S}}}{1-\phi } \end{array}$$

The osmotic pressure can be viewed as the energy to create a cavity with a unit volume in the crowder solution; the surface tension is the energy to create a unit-area interface between the crowder solution and a test protein; and the bending rigidity measures the energy arising from the curvature of the interface. Fitting our Δμ_e−v_ data to GFMT meant that we modeled the crowder proteins as spheres; in so doing we needed to specify the radius, *R*_c_, for each crowder protein. Note that *R*_c_ is the only free parameter; once *R*_c_ is chosen, the volume, surface area, and linear size of the test protein (i.e., *v*_p_, *s*_p_, and *l*_p_) are calculated by rolling a spherical probe of radius *R*_c_ around the three-dimensional structure of the test protein.

**Figure 3:**
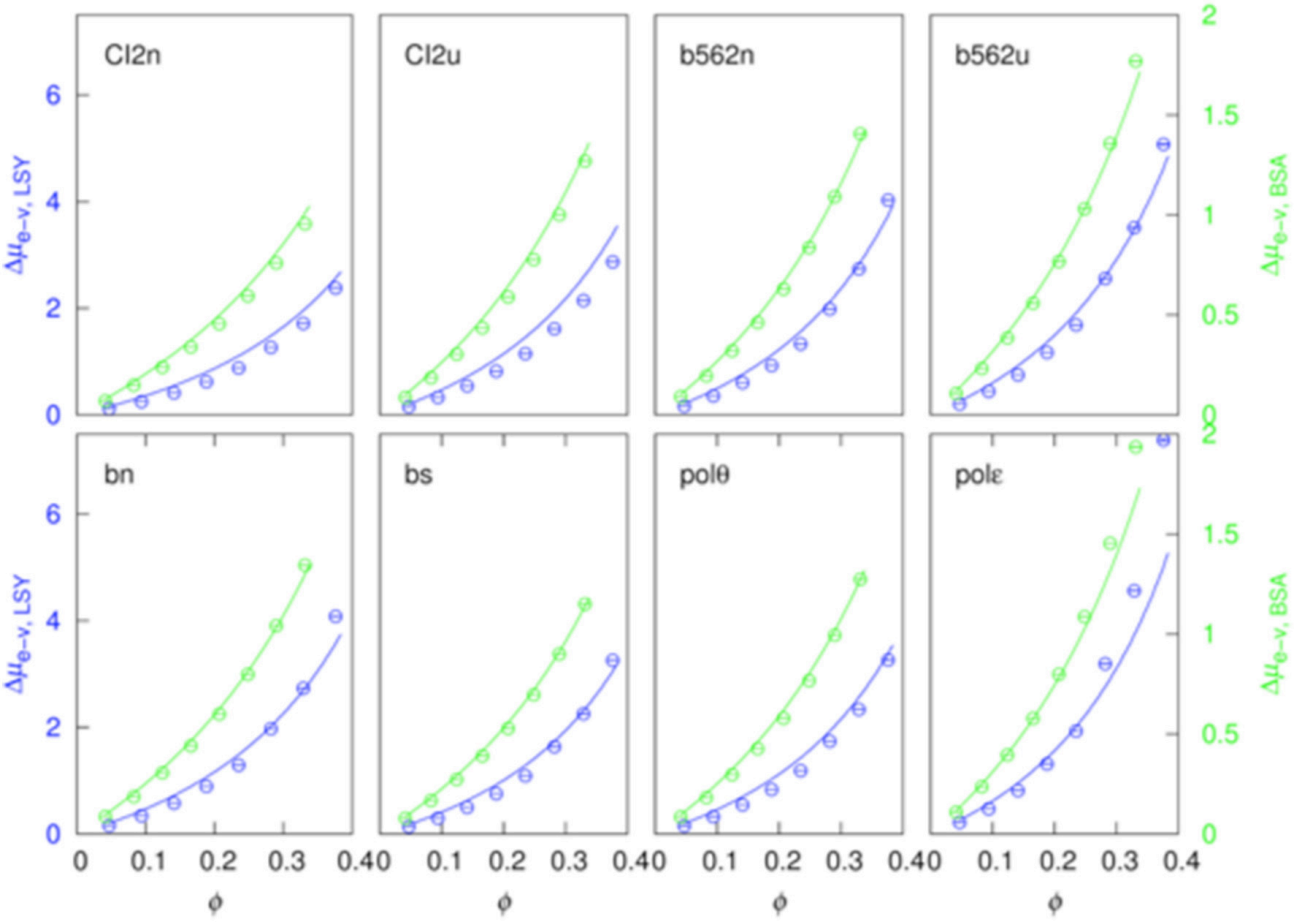
Fit of the excluded-volume component to the generalized fundamental measure theory.

We were able to achieve a good global fit for all the eight test proteins in either LYS or BSA after searching for an *R*_c_ value that minimized deviations between the Δμ_e−v_ data and the GFMT predictions (Figure 3). The resulting *R*_c_ values are 21.4 and 35.4 Å, respectively, for LYS and BSA. These values are close to the hydrodynamic radii, 19.6 and 36.5 Å, calculated by HYDROPRO (Ortega et al., 2011). Using the preceding *R*_c_ values, the volume fractions of the two crowders at their highest concentrations are 37.6% and 33.1%. The resulting values for *v*_p_, *s*_p_, and *l*_p_ of the eight test proteins are presented in Table S3.

### Quadratic Fitting of Soft-Attraction Component

**Figure 4:**
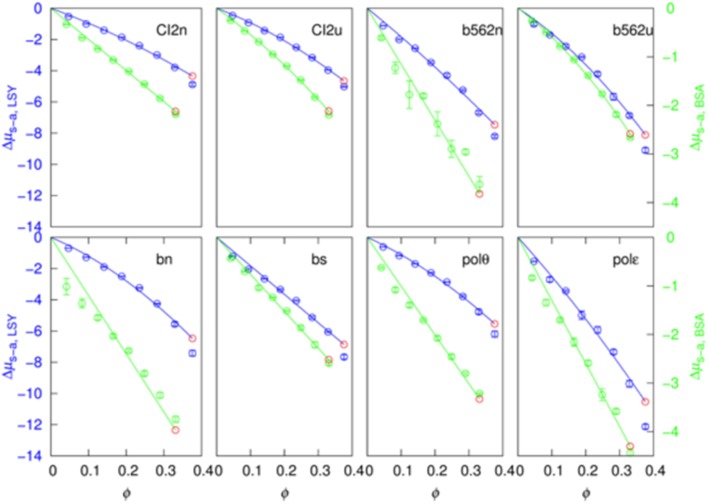
Fit of the soft-attraction component to a quadratic function of crowder volume fraction. The blue and green symbols are FMAP results, and the solid curves are fits using the first seven points. The predicted value at the eighth concentration is shown in red.

The soft-attraction component, Δμ_s−a_, calculated by FMAP has a nearly linear dependence on the crowder concentration (Figure 4). We fitted the results to a quadratic function:

(13) $$\begin{array}{l} \Delta \mu_{\text{s}-\text{a}}=a\phi +b\phi^{2} \end{array}$$

The quadratic term makes a minor contribution in most of the 16 sets of results (eight test proteins pairs with two crowder proteins). In particular, at ϕ = 30%, the quadratic term is <20% of the linear term in 9 out of the 16 cases.

Importantly, when the data at the first seven crowder concentrations were used for the fitting, the fitting function, by extrapolation, predicts well the Δμ_s−a_ result at the eighth crowder concentration (comparing the red circle and the eighth blue circle in each case). This suggests that a function like equation (15) can be used to predict Δμ_s−a_ at high crowder concentrations, where FMAP calculations become difficult because voids that can accommodate the test proteins are rare.

## Discussion

By using our FMAP, we have calculated the transfer energies of eight test proteins over a wide range of concentrations of two crowder proteins. We have shown that the transfer free energy can be accurately decomposed into an excluded-volume component, arising from the hard-core repulsion, and a soft-attraction component, arising from non-polar and electrostatic interactions. Our calculation results thus rigorously validate similar decompositions proposed previously (Petsev et al., 2003; Jiao et al., 2010; Minton, 2013). We have found that the excluded-volume component is predicted well by the generalized fundamental measure theory, which was developed for atomistic test proteins in the presence of spherical crowders that exert only steric repulsion. On the other hand, we have found that the soft-attraction component has a nearly linear dependence on crowder concentration. The latter result is interesting and has important implications.

Why does Δμ_s−a_ have a nearly linear dependence on crowder concentration? To gain insight, we turn to the perturbed virial expansion for pure molecular fluids (Nezbeda and Smith, 2004). The expansion was originally applied to the pressure (*P*),

(14) $$\begin{array}{l} \frac{P}{k_{\text{B}}T\rho }=\frac{P_{\text{ref}}}{k_{\text{B}}T\rho }+\underset{l\geq 2}{\sum }\Delta B_{l}\rho^{l-1} \end{array}$$

where ρ is the number density, *P*_ref_ is the pressure of a reference system, and Δ*B*_*l*_ are the “residual” virial coefficients, i.e., the differences in virial coefficients between the real and reference system. We can easily turn equation (14) into an expression for the excess chemical potential, using the relation (Qin and Zhou, 2016)

(15) $$\begin{array}{l} \mu^{\text{ex}}=\overset{\rho }{\underset{0}{\int }}\frac{1}{\rho }\frac{\partial P}{\partial \rho }d\rho \end{array}$$

The result is

(16) $$\begin{array}{l} \mu^{\text{ex}}=\mu_{\text{ref}}^{\text{ex}}+k_{\text{B}}T\underset{l\geq 2}{\sum }\left[l/\left(l-1\right)\right]B_{l}\rho^{l-1} \end{array}$$

A protein-crowder system where the test protein and crowder protein are the same is equivalent to a pure molecular fluid. In that case, the transfer free energy Δμ and the excess chemical potential μ^ex^ are equivalent. Furthermore, we may choose the reference system such that $\mu_{\text{ref}}^{\text{ex}}$ is equivalent to Δμ_e−v_, then the second term, an infinite sum and to be denoted as Δμ^ex^, on the right-hand side of equation (16) is equivalent to Δμ_s−a_. We can now recognize the quadratic function in equation (13) as a truncation of the infinite sum to the second order. The nearly linear dependence Δμ_s−a_ on crowder concentration just means that the contributions of the second and higher orders are much less than that of the first order.

In Figure S3, we display the contributions of the first-, second-, and third-order contributions to Δμ^ex^ for Lennard-Jones fluids, with the reference system chosen as hard-sphere fluids (diameter = σ). Over a wide range of ε, the depth of the interaction potential, the dominant contribution comes from the first order. Virial coefficients *B*_*l*_ are integrals of Mayer functions over the positions of *l* molecules. A Mayer function is the Boltzmann factor of the intermolecular interaction potential subtracted by 1; hence we expect that dominant contributions to residual virial coefficients Δ*B*_*l*_ come from clusters of *l* molecules in which all pairs are in the most attractive range of intermolecular distance. When the range of attraction is narrow, such molecular clusters become rare for *l* = 3 and higher. That would lead to small Δ*B*_*l*≥3_ values and explain why Δμ^ex^ is dominated by the first-order term, which is proportional to Δ*B*_2_. The foregoing argument for pure molecular fluids largely applies to the protein-crowder systems studied in the present work, thus providing a rationalization for the nearly linear dependence of Δμ_s−a_ on crowder concentration. An interesting future study would be to directly validate this argument by calculating virial coefficients of different orders for protein-crowder systems. It is straightforward to apply FMAP for *B*_2_ calculations, but efficient *B*_*l*≥3_ calculations for protein-crowder systems will require careful algorithmic design. Minton modeled soft attraction as weak unsaturable binding (Minton, 2013), which leads to an approximately linear dependence on ϕ. Hoppe and Minton (Hoppe and Minton, 2016) used a perturbed virial expansion, similar to equation (16) and including Δ*B*_2_ and Δ*B*_3_, for square-well crowders and found Δμ_s−a_ to be linearly dependent on ϕ. The present work provides confirmation of these previous results and generalize them to atomistic models.

A nearly linear dependence of Δμ_s−a_ allows us to extrapolate results obtained at lower crowder concentrations to higher ones, as we demonstrated here (Figure 4). As noted above, calculation of transfer free energies at high crowder concentrations becomes challenging for FMAP and likewise for other methods. For FMAP, the high percentage of fully clashed **Ω/c** combinations for the largest test protein studied here in the most concentrated LYS solution gives an indication of this challenge. A similar situation was observed in our previous study of disordered proteins in the presence of polyethylene glycol (Nguemaha et al., 2018). Extrapolation to higher crowder concentrations may provide a means to finesse this challenge.

While our interaction potential is atomistic, it is based on implicit solvent modeling. As a result, the treatment of hydration effects may prove inadequate, thereby limiting the accuracy of model predictions. In future studies we will investigate the performance of our atomistic model in quantitatively predicting experimental observables, including second virial coefficients.

As a final note, for determining the liquid-liquid phase equilibria of protein (Qin and Zhou, 2016) and colloid (Lomakin et al., 1996) solutions, it has been found that at least a third-order fitting is needed for the soft-attraction component of the excess chemical potential. In that case, a precise dependence of the chemical potential on protein concentration is key to determining the phase boundary, and hence one must calculate the chemical potential over as wide a range of protein concentration and cover the concentration range with as many points as can be done. For our protein-crowder systems, we find that, in a cubic fit, the second and third order terms have opposite signs and hence largely cancel each other, still leaving the first-order-term dominant.

## Author Contributions

VN conducted research and analyzed data. SQ prepared methods and engaged in discussion. H-XZ supervised research and wrote the manuscript.

### Conflict of Interest Statement

The authors declare that the research was conducted in the absence of any commercial or financial relationships that could be construed as a potential conflict of interest.

## Supplementary Material

Three figures comparing two methods of error estimates (Figure S1), showing non-additivity of the contributions to Δμ_s−a_ by the two types of soft interactions (Figure S2), and illustrating relatively small contributions to Δμ_s−a_ by higher order terms in a perturbed virial expansion of Δμ^*ex*^ (Figure S3); and three supporting tables, presenting data on transfer free energies (Tables S1,S2) and geometric properties (Table S3) of test proteins.
