# Supplementary material for: Transfer Free Energies of Test Proteins Into Crowded Protein Solutions Have Simple Dependence on Crowder Concentration
Source: Front Mol Biosci. 2019 May 29;6:39. doi: 10.3389/fmolb.2019.00039 (PMC6549383; doi:10.3389/fmolb.2019.00039)
Supplement: Supplementary file 1 [file Data_Sheet_1.PDF]

Transfer Free Energies of Test Proteins Into Crowded Protein Solutions Have Simple Dependence on Crowder Concentration

*Valery Nguemaha,[1] Sanbo Qin,[1] and Huan-Xiang Zhou[1,2,]**

[1]Department of Physics and Institute of Molecular Biophysics, Florida State University, Tallahassee, FL 32306, USA

[2]Department of Chemistry and Department of Physics, University of Illinois at Chicago, Chicago, IL 60607, USA

*Correspondence: hzhou43@uic.edu

Supporting Information

Table S1. Transfer free energies and components for eight test proteins under lysozyme crowding.

	CI2n	CI2u	b562n	b562u	bn	bs	polθ	polε
ϕ (%)	$\Delta\mu$ (kcal/mol)							
4.7	-0.41	-0.32	-0.95	-0.81	-0.54	-1.07	-0.47	-1.33
9.4	-0.75	-0.60	-1.65	-1.26	-0.94	-1.76	-0.85	-2.22
14.1	-1.00	-0.89	-1.96	-1.68	-1.32	-2.16	-1.15	-2.62
18.8	-1.24	-1.04	-2.54	-1.95	-1.61	-2.58	-1.44	-3.65
23.5	-1.52	-1.36	-2.97	-2.53	-1.95	-2.97	-1.67	-3.98
28.2	-1.73	-1.54	-3.25	-3.05	-2.27	-3.49	-2.03	-4.13
32.9	-2.07	-1.79	-3.85	-3.19	-2.74	-3.79	-2.39	-4.56
37.6	-2.48	-2.10	-4.00	-3.31	-3.24	-4.40	-2.97	-3.90
ϕ (%)	$\Delta\mu_{\mathrm{e-v}}$ (kcal/mol)							
4.7	0.11	0.15	0.16	0.20	0.16	0.14	0.15	0.21
9.4	0.25	0.33	0.36	0.44	0.34	0.30	0.32	0.47
14.1	0.41	0.54	0.60	0.75	0.58	0.50	0.55	0.82
18.8	0.62	0.82	0.93	1.17	0.89	0.76	0.83	1.31
23.5	0.88	1.15	1.33	1.68	1.29	1.09	1.19	1.93
28.2	1.27	1.61	1.99	2.56	1.97	1.64	1.74	3.19
32.9	1.71	2.14	2.74	3.51	2.73	2.25	2.34	4.56
37.6	2.38	2.87	4.03	5.07	4.08	3.26	3.26	7.71
ϕ (%)	$\Delta\mu_{\mathrm{s-a}}$ (kcal/mol)							
4.7	-0.53	-0.47	-1.12	-1.01	-0.70	-1.21	-0.62	-1.54
9.4	-1.00	-0.92	-2.00	-1.70	-1.28	-2.05	-1.17	-2.70
14.1	-1.41	-1.43	-2.56	-2.43	-1.90	-2.66	-1.70	-3.44
18.8	-1.86	-1.86	-3.47	-3.12	-2.50	-3.34	-2.27	-4.99
23.5	-2.40	-2.51	-4.30	-4.21	-3.24	-4.06	-2.86	-5.93
28.2	-3.00	-3.16	-5.24	-5.67	-4.25	-5.13	-3.78	-7.35
32.9	-3.79	-3.96	-6.69	-6.87	-5.56	-6.06	-4.76	-9.38
37.6	-4.88	-5.02	-8.20	-9.09	-7.42	-7.66	-6.19	-12.13

Table S2. Transfer free energies and components for eight test proteins under BSA crowding.

	CI2n	CI2u	b562n	b562u	bn	bs	polθ	polε
ϕ (%)	$\Delta\mu$ (kcal/mol)							
4.1	-0.26	-0.16	-0.52	-0.15	-0.93	-0.35	-0.54	-0.72
8.3	-0.46	-0.28	-1.03	-0.26	-1.17	-0.54	-0.90	-1.11
12.4	-0.60	-0.39	-1.46	-0.40	-1.35	-0.76	-1.10	-1.31
16.6	-0.73	-0.51	-1.34	-0.50	-1.59	-0.85	-1.28	-1.58
20.7	-0.84	-0.60	-1.75	-0.62	-1.73	-0.99	-1.50	-1.79
24.9	-0.97	-0.70	-2.06	-0.73	-2.00	-1.16	-1.69	-2.15
29.0	-1.10	-0.83	-1.87	-0.83	-2.21	-1.31	-1.81	-2.12
33.1	-1.23	-0.93	-2.20	-0.89	-2.39	-1.44	-1.94	-2.49
ϕ (%)	$\Delta\mu_{\mathrm{e-v}}$ (kcal/mol)							
4.1	0.07	0.09	0.09	0.11	0.09	0.08	0.09	0.11
8.3	0.15	0.19	0.20	0.23	0.19	0.17	0.18	0.24
12.4	0.24	0.30	0.32	0.38	0.31	0.27	0.30	0.40
16.6	0.34	0.44	0.46	0.56	0.44	0.39	0.43	0.58
20.7	0.46	0.59	0.63	0.77	0.60	0.53	0.58	0.80
24.9	0.59	0.78	0.84	1.03	0.80	0.70	0.77	1.09
29.0	0.76	1.00	1.09	1.36	1.04	0.90	0.99	1.45
33.1	0.96	1.27	1.41	1.77	1.34	1.15	1.27	1.94
ϕ (%)	$\Delta\mu_{\mathrm{s-a}}$ (kcal/mol)							
4.1	-0.33	-0.25	-0.61	-0.26	-1.01	-0.43	-0.62	-0.83
8.3	-0.61	-0.47	-1.23	-0.49	-1.36	-0.70	-1.08	-1.34
12.4	-0.84	-0.69	-1.78	-0.78	-1.66	-1.04	-1.39	-1.70
16.6	-1.07	-0.95	-1.81	-1.06	-2.03	-1.24	-1.70	-2.16
20.7	-1.30	-1.19	-2.38	-1.39	-2.33	-1.51	-2.08	-2.59
24.9	-1.56	-1.48	-2.90	-1.76	-2.80	-1.86	-2.46	-3.24
29.0	-1.86	-1.83	-2.96	-2.19	-3.25	-2.21	-2.80	-3.58
33.1	-2.18	-2.20	-3.62	-2.66	-3.75	-2.59	-3.21	-4.44

Table S3. Geometric properties of eight test proteins used by GFMT for predicting $\Delta\mu_{\mathrm{e-v}}$.

	R_c = 21.4 Å			R_c = 35.4 Å		
	l_p (Å)	s_p (Å^2)	v_p (Å^3)	l_p (Å)	s_p (Å^2)	v_p (Å^3)
CI2n	16.4	2956.4	12687.5	16.6	2981.3	13277.1
CI2u	23.0	4048.5	15884.1	23.5	4171.0	17335.5
b562n	22.1	4579.5	22255.3	22.6	4649.4	23778.0
b562u	26.2	5875.0	28863.9	27.1	6002.5	31602.8
bn	20.1	4367.6	22406.6	20.4	4437.5	23589.8
bs	17.9	3629.4	17926.4	18.1	3668.9	18641.3
polθ	20.5	4151.2	18241.0	20.9	4257.1	20026.8
polε	23.5	6362.7	39306.8	23.8	6440.1	41508.6

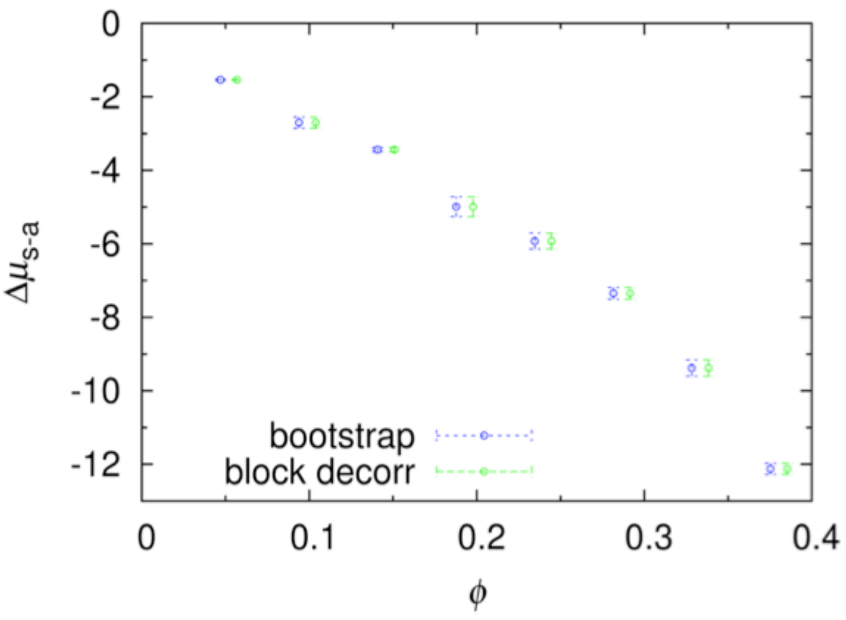

Fig. S1: Comparison of errors from two different methods, bootstrap and block decorrelation.

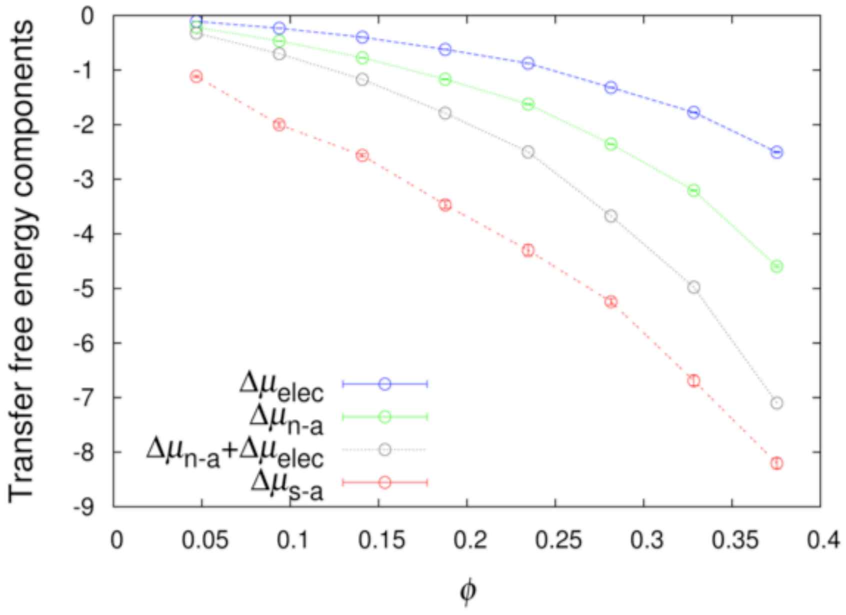

Fig. S2: Non-separability of the soft-attraction component. The sum of the contributions by the two types of soft interactions deviate significantly from the total soft-attraction component.

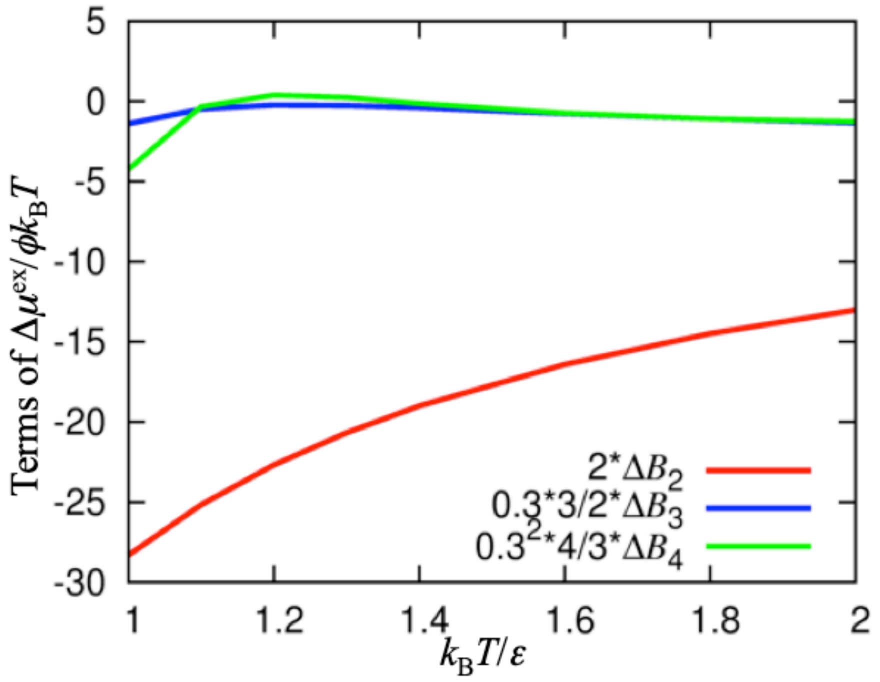

Fig. S3: Successive orders in the expansion of $\Delta\mu^{ex}$ for a Lennard-Jones fluid, at a relatively high volume fraction of ϕ = 30%.
